# Classification Models to Predict Survival of Kidney Transplant Recipients Using Two Intelligent Techniques of Data Mining and Logistic Regression

**Published:** 2017-05-01

**Authors:** M. Nematollahi, R. Akbari, S. Nikeghbalian, C. Salehnasab

**Affiliations:** 1 Anesthesiology and Critical Care Research Center, Shiraz University of Medical Sciences, Shiraz, Iran; 2 School of Computer Engineering & IT, Shiraz University of Technology, Shiraz, Iran; 3 School of Medicine, Shiraz University of Medical Sciences, Shiraz, Iran; 4 Shiraz University of Medical Sciences, Shiraz, Iran

**Keywords:** Kidney transplantation, Survival, Data mining, Neural networks, Support vector machine

## Abstract

Kidney transplantation is the treatment of choice for patients with end-stage renal disease (ESRD). Prediction of the transplant survival is of paramount importance. The objective of this study was to develop a model for predicting survival in kidney transplant recipients. In a cross-sectional study, 717 patients with ESRD admitted to Nemazee Hospital during 2008–2012 for renal transplantation were studied and the transplant survival was predicted for 5 years. The multilayer perceptron of artificial neural networks (MLP-ANN), logistic regression (LR), Support Vector Machine (SVM), and evaluation tools were used to verify the determinant models of the predictions and determine the independent predictors. The accuracy, area under curve (AUC), sensitivity, and specificity of SVM, MLP-ANN, and LR models were 90.4%, 86.5%, 98.2%, and 49.6%; 85.9%, 76.9%, 97.3%, and 26.1%; and 84.7%, 77.4%, 97.5%, and 17.4%, respectively. Meanwhile, the independent predictors were discharge time creatinine level, recipient age, donor age, donor blood group, cause of ESRD, recipient hypertension after transplantation, and duration of dialysis before transplantation. SVM and MLP-ANN models could efficiently be used for determining survival prediction in kidney transplant recipients.

## INTRODUCTION

Renal transplantation is the treatment of choice for end-stage renal disease (ESRD). Determination of graft survival is of paramount importance. In previous studies, classical statistical approaches were widely used for calculating the survival time. However, the methods used in those studies have many limitations in design and estimation [1-3]. The increasing use of new techniques of data mining, especially for discovering new patterns, has become more common and routine in medical sciences. Data mining techniques can help us predict the survival time of kidney transplants [4].

Nowadays, data mining techniques are of great popularity in the modeling of medical data [4, 5]. The first technique is multilayer perceptron of artificial neural networks (MLP-ANN) that is a feed-forward with one or more layers between the input and output layer. MLPs are widely used for prediction, recognition, pattern classification, and approximation of data [6]. In this regard, Petrovsky and Brier used neural network techniques to predict transplant outcomes [7, 8]. The second technique is support vector machine (SVM), another popular and powerful data mining classification technique in machine learning [9-11]. This technique works well with noisy data [12]. It was used by Yang and Yahav to analyze the transplantation survival [13, 14].

In most previous studies one intelligence method was compared with a classical method [15-17]. Therefore, the objective of this study was to use data-mining techniques to predict kidney transplantation survival for patients transplanted at Nemazee Hospital, Shiraz, southern Iran, between 2008 and 2012, by comparing two types of more frequently used intelligence methods in data-mining area with logistic regression.

## MATERIALS AND METHODS

The participants included 717 transplant recipients with 24 attributes operated at Nemazee Hospital, Shiraz, southern Iran between 2008 and 2012. Incomplete records were excluded in the primary phase of the study. A researcher-made questionnaire was used for collecting the required data. The study variables, categorized into three main groups, included recipient variables (blood group, Rh, hypertension after transplantation, use of immunosuppressive drugs (Sandimmune Neoral, Prograf, CellCept, methylprednisolone, prednisone, and thymoglobulin), duration of dialysis before transplantation, cause of ESRD, sex, age, weight, and serum creatinine level at the time of discharge); donor variables (blood group, Rh, sex, age, and type of donor); and transplantation variables (cold storage time).

For data collection, the patient`s medical records were reviewed and data were extracted from them; then we followed other survival data with hospital software, dialysis centers, and telephone contact with her/his family. Finally, 717 files of kidney recipients were selected as the sample of the study.

The current study tried to predict the 5-year survival in kidney transplant recipients—602 (84.0%) who survived and 115 (16.0%) who died within five years of transplantation. IBM SPSS Modeler was used for pre-processing, modeling, and evaluating data using a global standard CRISP-DM [18].

In the pre-processing stage, we replaced missing values of continuous variables with mean and those of categorical variables with mode of data. The variables of recipient height and cold storage time were excluded because they were missed in more than half of the records.

After pre-processing, there were two more phases—modeling made and evaluating the results. In the first phase, all entrance variables were used in modeling. In the next phase, the entrance variables, the independent predictors identified by at least two models based on AUC, accuracy, sensitivity, and specificity of the models in phase 1, the experts opinion, and clinical findings, were used in the selected models as input. 

After modeling, three measurement criteria (accuracy, sensitivity, and specificity) and AUC were used to evaluate the models.

## Results

The mortality rate attributed to transplantation was 4.6%. The results of modeling and independent predictors are presented in Tables 1 and 2. The SVM model had the highest accuracy of 90.4%. The four independent predictors, discharge time creatinine level, recipient age, and donor blood group and age, had significant occurrence in all three tested models (Table 2).

**Table 1:** Ranking models based on the measurement criteria

Rank number	Model name used	Accuracy%	Area under curve%	Sensitivity%	Specificity%
1	SVM	90.4	86.5	98.2	49.6
2	MLP ANN	85.9	76.9	97.3	26.1
3	LR	84.7	77.4	97.5	17.4

**Table 2:** The independent predictors of renal transplant recipients survival

Predictor	Technique			Occurrence
	LR	SVM	MLP ANN	
Discharge time creatinine	●	●	●	3
Recipient age	●	●	●	3
Donor age	●	●	●	3
Donor blood group	●	●	●	3
Recipient hypertension after transplantation		●	●	2
Cause of ESRD	●		●	2
Duration of dialysis before transplantation	●		●	2

## DISCUSSION

We found SVM, MLP-ANN, and LR the most appropriate models for prediction of renal transplant recipient survival. Hoot [19] conducted a study to predict the graft survival rate of liver transplant recipients. The main limitation of this study was that it used only a small number of variables and it had only 67% accuracy. Brier [8] also found an overall prediction accuracy of 64% for LR and 63% for MLP-ANN. ANNs were most closely related to LR results for prediction and discriminant analysis for classification. In that study, only one factor, transplantation of kidney from a white donor to black recipient, was associated with a statistically significant risk factor [8]. MLP-ANN predictors have been shown to offer a more flexible modeling environment than other statistical methods [20].

In general, determining the accuracy of the predictive models to predict particular medical issues is very complicated. This complexity can be caused by factors such as lack of collecting critical data in appropriate time and location. Many previous studies in the area of survival predictions have been performed using different statistical techniques and ANN, which is a subset of the data mining techniques. Neural networks are one of the most widely used techniques in the field of medical survey data [4, 8, 20]. In this study, after SVM, the MLP-ANN model with an accuracy of 85.9%, was suitable for predicting the survival of transplantation. The results of this study were consistent with those of another study [7] with an accuracy of 78.5%. The SVM is another model based on the accuracy discussed in this study. The prediction accuracy of this model was higher than other models used. One of the reasons was that this method is a good technique to differentiate samples or boundary points. A study [13] demonstrated the usefulness of this technique in predicting survival, but the accuracy of the model has not been mentioned.

In conclusion, SVM and MLP-ANN models can efficiently be used to predict renal transplant recipient survival. Discharge time creatinine level, recipient age, donor age, donor blood group, cause of ESRD, recipient hypertension after transplantation, and duration of dialysis before transplantation were independent predictors for survival of kidney transplant recipients. Attention to the condition of dialysis before transplantation, control of high blood pressure at the discharge time and the cause of ESRD could efficiently be used for determining survival prediction in kidney transplant recipients. The results of this study were comparable with those from statistical models.
